# Supplementary material for: Material-agnostic machine learning approach enables high relative density in powder bed fusion products
Source: Nat Commun. 2023 Oct 17;14:6557. doi: 10.1038/s41467-023-42319-x (PMC10582079; doi:10.1038/s41467-023-42319-x)
Supplement: Supplementary file 1 — Supplementary Information [file 41467_2023_42319_MOESM1_ESM.pdf]

Supplementary Information

Material-Agnostic Machine Learning Approach Enables High Relative Density in Powder Bed Fusion Products

Jaemin Wang[1], Sang Guk Jeong[1], Eun Seong Kim[1], Hyoung Seop Kim[2, 3, 4, 5], and Byeong-Joo Lee[1,*]

[1] Department of Materials Science and Engineering,
Pohang University of Science and Technology (POSTECH), Pohang 37673, Republic of Korea

[2] Graduate Institute of Ferrous and Energy materials Technology (GIFT), Pohang University of Science and Technology (POSTECH), Pohang 37673, Republic of Korea

[3] Center for Heterogenic Metal Additive Manufacturing, Pohang University of Science and Technology (POSTECH), Pohang 37673, Republic of Korea

[4] Institute for Convergence Research and Education in Advanced Technology, Yonsei University, Seoul, 03722, Republic of Korea

[5] Advanced Institute for Materials Research (WPI-AIMR), Tohoku University, Sendai, 980-8577, Japan

Supplementary Notes

Detailed information about the XGBoost model and the other models.

Before the hyperparameter optimization of Random Forest and XGBoost models, all models used default hyperparameters written as below.

Random Forest:

- Trees: 100 (n_estimators=100)
- Maximum Tree Depth: Unlimited (max_depth=None)
- Minimum Samples for Node Split: 2 (min_samples_split=2)
- Minimum Samples at Leaf Node: 1 (min_samples_leaf=1)
- Bootstrapping: Yes (bootstrap=True)

XGBoost:

- Learning Rate: 0.3 (eta=0.3)
- Maximum Tree Depth: 6 (max_depth=6)
- Minimum Child Weight: 1 (min_child_weight=1)

K-Neighbors:

- Neighbors: 5 (n_neighbors=5)
- Weight Scheme: Uniform (weights='uniform')

Decision Tree:
- Maximum Tree Depth: Unlimited (max_depth=None)
- Minimum Samples for Node Split: 2 (min_samples_split=2)
- Minimum Samples at Leaf Node: 1 (min_samples_leaf=1)

Multi-Layer Perceptron:
- Activation Function: ReLU (activation='relu')
- Solver: 'adam'
- Regularization: None (alpha=0.0001)
- Hidden Layers: 2 with 100 neurons each

Support Vector Machine:
- Kernel: Radial Basis Function (kernel='rbf')
- Regularization Term (C): 1.0 (C=1.0)
- Epsilon: 0.1 (epsilon=0.1)

Stochastic Gradient Descent:
- Loss: Squared loss (loss='squared_loss')
- Penalty: 'l2' with strength 0.0001 (alpha=0.0001)

Ridge:
- Regularization: L2 with strength 1.0 (alpha=1.0)
- Compute Intercept: Yes (fit_intercept=True)

Linear Regression:
- Method: Ordinary Least Squares
- Compute Intercept: Yes (fit_intercept=True)

Ridge CV:
- Ridge regression with built-in cross-validation
- Regularization Strength: Determined by cross-validation (alpha)

Bayesian Ridge:
- Default Regularization Parameters:
- alpha_1=1e-06,
- alpha_2=1e-06,
- lambda_1=1e-06,
- lambda_2=1e-06

Lasso CV:

- Lasso regression with built-in cross-validation
- Regularization Strength: Determined by cross-validation (alpha)

Elastic Net CV:

- Elastic Net regression with built-in cross-validation
- Regularization Strength and L1 Ratio: Determined by cross-validation

Gaussian Process Regression:

- Kernel: 1.0 RBF (kernel=1.0 * RBF(1.0))

Partial Least Squared Regression (PLS):

- Components: 2 (n_components=2)

Lasso:

- Regularization: L1 with strength 1.0 (alpha=1.0)
- Compute Intercept: Yes (fit_intercept=True)

Elastic Net:

- Regularization: Mix of L1 and L2
- Weighting: Equal L1 and L2 (l1_ratio=0.5)
- Combined Strength: 1.0 (alpha=1.0)

Kernel Ridge:

- Kernel: Linear (kernel='linear')
- Regularization Strength: 1 (alpha=1)

After the hyperparameter optimization using Optuna, the XGBoost model and the Random Forest Model used hyperparameters written as below. Both models used early stopping method.

XGBoost:

- Max Depth: 15 (max_depth=15)
- Learning Rate: 0.0272 (learning_rate=0.0272)
- Trees: 8800 (n_estimators=8800)
- Column Sample by Tree: 1.0 (colsample_bytree=1.0)
- Column Sample by Level: 0.63 (colsample_bylevel=0.63)
- Column Sample by Node: 0.67 (colsample_bynode=0.67)
- Lambda: 0.03 (reg_lambda=0.03)
- Alpha: 0.01 (reg_alpha=0.01)
- Subsample: 0.75 (subsample=0.75)
- Minimum Child Weight: 2 (min_child_weight=2)

Random Forest:

- Trees: 144 (n_estimators=144)
- Maximum Depth: 33 (max_depth=33)
- Minimum Samples for Node Split: 2 (min_samples_split=2)
- Minimum Samples at Leaf Node: 1 (min_samples_leaf=1)
- Bootstrapping: Yes (bootstrap=True)

The references to the data for machine learning model training and validation and Fig. 1.

The references are presented in here[1-118].

Supplementary Tables

Supplementary Table 1. STS 316L powder data from the training dataset for the range where the process conditions in Table 3 exist.

#	Laser Power [W]	Scan Speed [mm/s]	Layer Thickness [mm]	Hatch Spacing [mm]	Relative Density [%]
1	350	650	0.05	0.11	99.60
2	380	950	0.05	0.08	99.18
3	380	850	0.05	0.09	99.18
4	380	700	0.05	0.11	99.15
5	380	750	0.05	0.1	99.14
6	380	625	0.05	0.12	99.12
7	180	694.73	0.05	0.124	91.15

Supplementary Table 2. Process condition prediction results for AlSi10Mg powder and their respective relative densities of the manufactured specimens measured through experiments.

#	Laser Power (W)	Scan Speed (mm/s)	Layer Thickness (mm)	Hatch Spacing (mm)	Relative Density (%)
1	398	2150	0.05	0.081	0.053<br>0.07<br>0.084
2	343	1600	0.05	0.105	0.065<br>0.102<br>0.142
3	314	1370	0.05	0.119	0.105<br>0.114<br>0.14
4	391	1540	0.05	0.083	0.105<br>0.161
5	322	1320	0.05	0.08	0.212<br>0.233
6	397	1470	0.05	0.118	0.251<br>0.338
7	272	1260	0.05	0.143	0.226<br>0.314<br>0.358
8	368	1250	0.05	0.097	0.418<br>0.446
9	236	990	0.05	0.131	0.442<br>0.477<br>0.651
10	278	1230	0.05	0.098	0.544<br>0.589
11	321	980	0.05	0.104	0.601<br>0.688<br>0.824
12	362	1270	0.05	0.148	1.238<br>2.514

Supplementary Table 3. AlSi10Mg powder data from the training dataset for the range where the process conditions in Table 4 exist.

#	Laser Power [W]	Scan Speed [mm/s]	Layer Thickness [mm]	Hatch Spacing [mm]	Relative Density [%]
1	300	1270	0.045	0.08	99.78
2	250	1100	0.06	0.13	99.50
3	250	1200	0.06	0.13	99.50
4	250	1000	0.06	0.13	99.30
5	250	1500	0.06	0.105	98.90
6	250	1400	0.06	0.13	98.50
7	250	1600	0.06	0.13	96.90
8	250	1800	0.06	0.13	94.50

Supplementary Table 4. Process condition prediction results for $Fe_{60}Co_{15}Ni_{15}Cr_{10}$ MEA powder and their respective relative densities of the manufactured specimens measured through experiments.

#	Laser Power (W)	Scan Speed (mm/s)	Layer Thickness (mm)	Hatch Spacing (mm)	Relative Density (%)
1	302	980	0.05	0.103	99.971<br>99.964
2	365	1010	0.05	0.118	99.886<br>99.881<br>99.934<br>99.907
3	282	780	0.05	0.122	99.908<br>99.920<br>99.876
4	349	1070	0.05	0.087	99.872<br>99.924
5	367	740	0.05	0.156	99.950<br>99.809<br>99.823
6	399	840	0.05	0.102	99.887<br>99.825
7	244	790	0.05	0.098	99.846<br>99.913<br>99.763
8	397	920	0.05	0.14	99.885<br>99.823<br>99.785
9	289	540	0.05	0.151	99.835<br>99.777<br>99.713
10	373	530	0.05	0.183	99.628<br>99.695<br>99.751
11	392	550	0.05	0.219	99.545<br>99.703<br>99.699
12	385	1380	0.05	0.094	99.119<br>99.281<br>98.790

Supplementary Figures

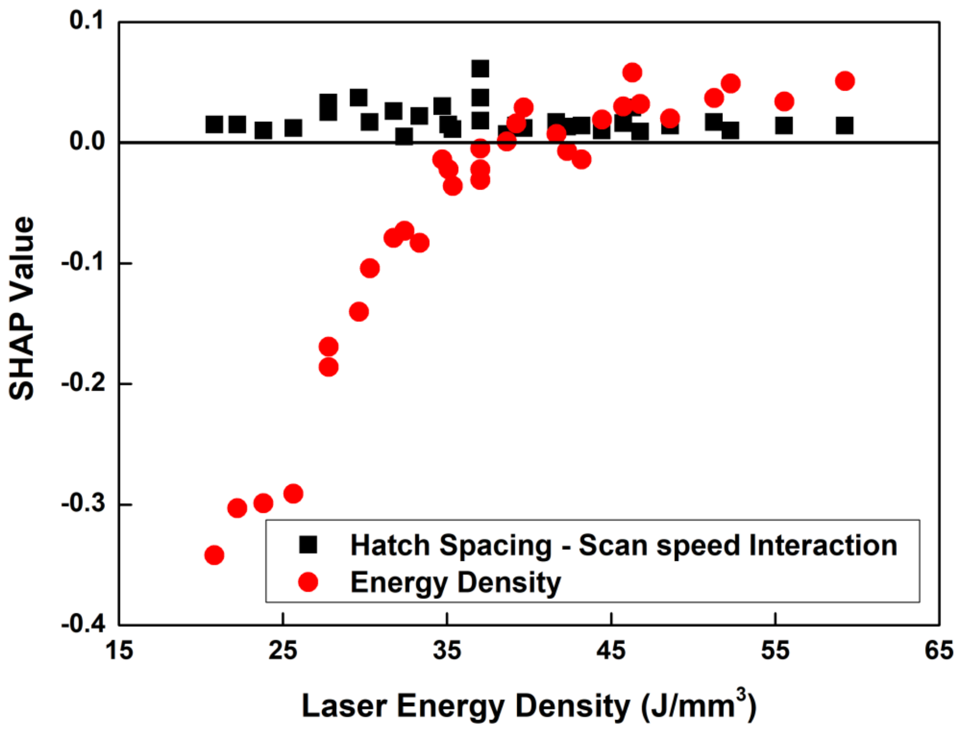

Supplementary Figure 1. **Laser Energy Density Versus SHAP Score for STS 316L Under Specific Process Conditions.** This figure illustrates the relationship between laser energy density and SHAP score when processing STS 316L with a hatch distance of 0.15 mm and a scan speed equal to or greater than 1200 mm/s. Source data are provided as a Source Data file.

Supplementary References

1. Liu, X., Liu, Y., Zhou, Z., Zhong, H. & Zhan, Q. A combination strategy for additive manufacturing of AA2024 high-strength aluminium alloys fabricated by laser powder bed fusion: Role of hot isostatic pressing. *Mater. Sci. Eng. A* **850**, 143597 (2022).
2. Qi, Y. *et al.* A high strength Al–Li alloy produced by laser powder bed fusion: Densification, microstructure, and mechanical properties. *Addit. Manuf.* **35**, 101346 (2020).
3. Tonelli, L., Ahmed, M. M. Z. & Ceschini, L. A novel heat treatment of the additively manufactured Co28Cr6Mo biomedical alloy and its effects on hardness, microstructure and sliding wear behavior. *Prog. Addit. Manuf.* **8**, 313–329 (2022).
4. Liu, Y., Li, M., Lu, X., Zhu, X. & Li, P. A Novel Required Laser Energy Predicting Model for Laser Powder Bed Fusion. *Metals (Basel).* **11**, 1966 (2021).
5. Tang, X. *et al.* A study on the mechanical and electrical properties of high-strength CuCrZr alloy fabricated using laser powder bed fusion. *J. Alloys Compd.* **924**, 166627 (2022).
6. Carrozza, A., Aversa, A., Fino, P. & Lombardi, M. A study on the microstructure and mechanical properties of the Ti-6Al-2Sn-4Zr-6Mo alloy produced via Laser Powder Bed Fusion. *J. Alloys Compd.* **870**, 159329 (2021).
7. Qu, S., Ding, J. & Song, X. Achieving Triply Periodic Minimal Surface Thin-Walled Structures by Micro Laser Powder Bed Fusion Process. *Micromachines* **12**, 705 (2021).
8. Mehta, A. *et al.* Additive manufacturing and mechanical properties of the dense and crack free Zr-modified aluminum alloy 6061 fabricated by the laser-powder bed fusion. *Addit. Manuf.* **41**, 101966 (2021).
9. Lu, B. Additive Manufacturing of Copper-based Alloy by Laser Powder Bed Fusion. *Electronic Theses and Dissertations, 2020-* (2020).
10. Hyer, H. *et al.* Additive manufacturing of dense WE43 Mg alloy by laser powder bed fusion. *Addit. Manuf.* **33**, 101123 (2020).
11. Zhang, B. *et al.* An efficient framework for printability assessment in Laser Powder Bed Fusion metal additive manufacturing. *Addit. Manuf.* **46**, 102018 (2021).
12. Åhman, H. N., Thorsson, L., Mellin, P., Lindwall, G. & Persson, C. An Enhanced Understanding of the Powder Bed Fusion–Laser Beam Processing of Mg-Y3.9wt%-Nd3wt%-Zr0.5wt% (WE43) Alloy through Thermodynamic Modeling and Experimental Characterization. *Materials (Basel).* **15**, 417 (2022).
13. Gong, H., Rafi, K., Gu, H., Starr, T. & Stucker, B. Analysis of defect generation in Ti–6Al–4V parts made using powder bed fusion additive manufacturing processes. *Addit. Manuf.* **1–4**, 87–98 (2014).
14. Attarzadeh, F. & Asadi, E. Analysis of element loss, densification, and defects in laser-based powder-bed fusion of magnesium alloy WE43. *J. Magnes. Alloy.* **10**, 2118–2136 (2022).
15. Sun, Z. H. *et al.* Assessing Influencing Factors of Residual Stresses in SLM using a Novel Analysis Method. *Proc. 16th Int. Symp. Electromachining* **20**, 531–537 (2010).
16. Stugelmayer, E. J. Characterization of process induced defects in laser powder bed fusion processed AlSi10Mg alloy. (2018).
17. Hastie, J. C., Kartal, M. E., Carter, L. N., Attallah, M. M. & Mulvihill, D. M. Classifying shape of internal pores within AlSi10Mg alloy manufactured by laser powder bed fusion using 3D X-ray micro computed tomography: Influence of processing parameters and heat treatment. *Mater. Charact.* **163**, 110225 (2020).
18. Spierings, A. B., Schneider, M. & Eggenberger, R. Comparison of density measurement techniques for additive manufactured metallic parts. *Rapid Prototyp. J.* **17**, 380–386 (2011).
19. Ahmed Obeidi, M. *et al.* Comparison of the porosity and mechanical performance of 316L stainless steel manufactured on different laser powder bed fusion metal additive manufacturing machines. *J. Mater. Res. Technol.* **13**, 2361–2374 (2021).
20. Hyer, H. *et al.* Composition-dependent solidification cracking of aluminum-silicon alloys during laser powder bed fusion. *Acta Mater.* **208**, 116698 (2021).

21. Zou, J. *et al.* Controlling the grain orientation during laser powder bed fusion to tailor the magnetic characteristics in a Ni-Fe based soft magnet. *Acta Mater.* **158**, 230–238 (2018).
22. Niu, X., Shen, H., Fu, J., Yan, J. & Wang, Y. Corrosion behaviour of laser powder bed fused bulk pure magnesium in hank's solution. *Corros. Sci.* **157**, 284–294 (2019).
23. Suchý, J. *et al.* Corrosion behaviour of WE43 magnesium alloy printed using selective laser melting in simulation body fluid solution. *J. Manuf. Process.* **69**, 556–566 (2021).
24. Salvan, C., Briottet, L., Baffie, T., Guetaz, L. & Flament, C. CuCrZr alloy produced by laser powder bed fusion: Microstructure, nanoscale strengthening mechanisms, electrical and mechanical properties. *Mater. Sci. Eng. A* **826**, 141915 (2021).
25. Gordon, J. V. *et al.* Defect structure process maps for laser powder bed fusion additive manufacturing. *Addit. Manuf.* **36**, 101552 (2020).
26. Kamath, C., El-Dasher, B., Gallegos, G. F., King, W. E. & Sisto, A. Density of additively-manufactured, 316L SS parts using laser powder-bed fusion at powers up to 400 W. *Int. J. Adv. Manuf. Technol.* **74**, 65–78 (2014).
27. Julmi, S. *et al.* Development of a Laser Powder Bed Fusion Process Tailored for the Additive Manufacturing of High-Quality Components Made of the Commercial Magnesium Alloy WE43. *Materials (Basel).* **14**, 887 (2021).
28. Ghodsi, M. Z. *et al.* Development of Yttria-stabilized zirconia reinforced Inconel 625 metal matrix composite by laser powder bed fusion. *Mater. Sci. Eng. A* **827**, 142037 (2021).
29. Larimian, T., Kannan, M., Grzesiak, D., AlMangour, B. & Borkar, T. Effect of energy density and scanning strategy on densification, microstructure and mechanical properties of 316L stainless steel processed via selective laser melting. *Mater. Sci. Eng. A* **770**, 138455 (2020).
30. Shen, F. L., Li, H. Q., Guo, H., Guo, N. N. & Fang, X. Y. Effect of energy density on the superelastic property of Ni-rich NiTi alloy fabricated by laser powder bed fusion. *Mater. Sci. Eng. A* **854**, 143874 (2022).
31. Wei, K., Gao, M., Wang, Z. & Zeng, X. Effect of energy input on formability, microstructure and mechanical properties of selective laser melted AZ91D magnesium alloy. *Mater. Sci. Eng. A* **611**, 212–222 (2014).
32. Wallis, C. & Buchmayr, B. Effect of heat treatments on microstructure and properties of CuCrZr produced by laser-powder bed fusion. *Mater. Sci. Eng. A* **744**, 215–223 (2019).
33. Wang, P., Salandari-Rabori, A., Dong, Q. & Fallah, V. Effect of input powder attributes on optimized processing and as-built tensile properties in laser powder bed fusion of AlSi10Mg alloy. *J. Manuf. Process.* **64**, 633–647 (2021).
34. Eliasu, A., Czekanski, A. & Boakye-Yiadom, S. Effect of laser powder bed fusion parameters on the microstructural evolution and hardness of 316L stainless steel. *Int. J. Adv. Manuf. Technol.* **113**, 2651–2669 (2021).
35. Ramachandiran, N. *et al.* Effect of non-lamellar α precipitate morphology on the mechanical properties of Ti5553 parts made by laser powder-bed fusion at high laser scan speeds. *Mater. Sci. Eng. A* **841**, 143039 (2022).
36. Leicht, A., Rashidi, M., Klement, U. & Hryha, E. Effect of process parameters on the microstructure, tensile strength and productivity of 316L parts produced by laser powder bed fusion. *Mater. Charact.* **159**, 110016 (2020).
37. Sidambe, A. T., Tian, Y., Prangnell, P. B. & Fox, P. Effect of processing parameters on the densification, microstructure and crystallographic texture during the laser powder bed fusion of pure tungsten. *Int. J. Refract. Met. Hard Mater.* **78**, 254–263 (2019).
38. Hovig, E. W., Holm, H. D. & Sørby, K. Effect of processing parameters on the relative density of alsi10mg processed by laser powder bed fusion. *Lect. Notes Electr. Eng.* **484**, 268–276 (2019).
39. Liverani, E., Toschi, S., Ceschini, L. & Fortunato, A. Effect of selective laser melting (SLM) process parameters on microstructure and mechanical properties of 316L austenitic stainless steel. *J. Mater. Process. Technol.* **249**, 255–263 (2017).
40. Dadbakhsh, S., Hao, L. & Sewell, N. Effect of selective laser melting layout on the quality of stainless steel parts. *Rapid Prototyp. J.* **18**, 241–249 (2012).

41. Sing, S. L., Huang, S. & Yeong, W. Y. Effect of solution heat treatment on microstructure and mechanical properties of laser powder bed fusion produced cobalt-28chromium-6molybdenum. *Mater. Sci. Eng. A* **769**, 138511 (2020).
42. Niu, X., Shen, H., Fu, J. & Feng, J. Effective control of microstructure evolution in AZ91D magnesium alloy by SiC nanoparticles in laser powder-bed fusion. *Mater. Des.* **206**, 109787 (2021).
43. Fan, H. & Yang, S. Effects of direct aging on near-alpha Ti–6Al–2Sn–4Zr–2Mo (Ti-6242) titanium alloy fabricated by selective laser melting (SLM). *Mater. Sci. Eng. A* **788**, 139533 (2020).
44. Yamamoto, T., Hara, M. & Hatano, Y. Effects of fabrication conditions on the microstructure, pore characteristics and gas retention of pure tungsten prepared by laser powder bed fusion. *Int. J. Refract. Met. Hard Mater.* **95**, 105410 (2021).
45. Watring, D. S., Benzing, J. T., Hrabe, N. & Spear, A. D. Effects of laser-energy density and build orientation on the structure–property relationships in as-built Inconel 718 manufactured by laser powder bed fusion. *Addit. Manuf.* **36**, 101425 (2020).
46. Obeidi, M. A. *et al.* Effects of powder compression and laser re-melting on the microstructure and mechanical properties of additively manufactured parts in laser-powder bed fusion. *Results Mater.* **13**, 100264 (2022).
47. Lv, J., Shen, H. & Fu, J. Effects of the process parameters on the formability and properties of Ni54(at.%) Ti alloys prepared by laser powder bed fusion. *Rapid Prototyp. J.* **28**, 1193–1205 (2022).
48. Nandy, J., Sahoo, S., Sarangi, H. & Sabat, R. K. Evaluation of structural and mechanical properties of high strength aluminum alloy components fabricated using laser powder bed fusion process. *J. Laser Appl.* **33**, 032009 (2021).
49. Schmitt, M., Schlick, G., Seidel, C. & Reinhart, G. Examination of the processability of 16MnCr5 by means of laser powder bed fusion. *Procedia CIRP* **74**, 76–81 (2018).
50. Lee, S. H. W., Choo, H. L., Mok, S. H., Cheng, X. Y. & Manurung, Y. H. P. Fabrication of porous metallic materials by controlling the processing parameters in selective laser melting process. *AIP Conf. Proc.* **2233**, (2020).
51. Vallejo, N. D., Kljestan, N., Ayers, N., Knezevic, M. & Sohn, Y. Flaw type dependent tensile properties of 316L stainless steel additively manufactured by laser powder bed fusion. *Results Mater.* **15**, 100315 (2022).
52. Li, J., Qu, H. & Bai, J. Grain boundary engineering during the laser powder bed fusion of TiC/316L stainless steel composites: New mechanism for forming TiC-induced special grain boundaries. *Acta Mater.* **226**, 117605 (2022).
53. Wen, S. *et al.* High-density tungsten fabricated by selective laser melting: Densification, microstructure, mechanical and thermal performance. *Opt. Laser Technol.* **116**, 128–138 (2019).
54. Qu, S. *et al.* High-precision laser powder bed fusion processing of pure copper. *Addit. Manuf.* **48**, 102417 (2021).
55. Liu, J. *et al.* Hot cracking in ZK60 magnesium alloy produced by laser powder bed fusion process. *Mater. Lett.* **301**, 130283 (2021).
56. Ghasemi, A. *et al.* Influence of alloying elements on laser powder bed fusion processability of aluminum: A new insight into the oxidation tendency. *Addit. Manuf.* **46**, 102145 (2021).
57. Wei, K., Wang, Z. & Zeng, X. Influence of element vaporization on formability, composition, microstructure, and mechanical performance of the selective laser melted Mg–Zn–Zr components. *Mater. Lett.* **156**, 187–190 (2015).
58. Pan, T. Influence of Input Energy on Mechanical Properties of Laser Powder Bed Fused Aisi 304l Stainless Steel. (2020).
59. Liu, S. *et al.* Influence of laser process parameters on the densification, microstructure, and mechanical properties of a selective laser melted AZ61 magnesium alloy. *J. Alloys Compd.* **808**, 151160 (2019).
60. Delgado, J., Ciurana, J. & Rodríguez, C. A. Influence of process parameters on part quality and mechanical properties for DMLS and SLM with iron-based materials. *Int. J. Adv. Manuf. Technol.* **60**, 601–610 (2012).

61. Calignano, F. & Minetola, P. Influence of Process Parameters on the Porosity, Accuracy, Roughness, and Support Structures of Hastelloy X Produced by Laser Powder Bed Fusion. *Materials (Basel).* **12**, 3178 (2019).
62. Ahmed, M., Obeidi, M. A., Yin, S. & Lupoi, R. Influence of processing parameters on density, surface morphologies and hardness of as-built Ti-5Al-5Mo-5V-3Cr alloy manufactured by selective laser melting. *J. Alloys Compd.* **910**, 164760 (2022).
63. Dilip, J. J. S. *et al.* Influence of processing parameters on the evolution of melt pool, porosity, and microstructures in Ti-6Al-4V alloy parts fabricated by selective laser melting. *Prog. Addit. Manuf.* **2**, 157–167 (2017).
64. Cherry, J. A. *et al.* Investigation into the effect of process parameters on microstructural and physical properties of 316L stainless steel parts by selective laser melting. *Int. J. Adv. Manuf. Technol.* **76**, 869–879 (2015).
65. Wang, D., Song, C., Yang, Y. & Bai, Y. Investigation of crystal growth mechanism during selective laser melting and mechanical property characterization of 316L stainless steel parts. *Mater. Des.* **100**, 291–299 (2016).
66. Tucho, W. M., Lysne, V. H., Austbø, H., Sjolyst-Kverneland, A. & Hansen, V. Investigation of effects of process parameters on microstructure and hardness of SLM manufactured SS316L. *J. Alloys Compd.* **740**, 910–925 (2018).
67. Liu, B., Wildman, R., Tuck, C., Ashcroft, I. & Hague, R. Investigation the Effect of Particle Size Distribution on Processing Parameters Optimisation in Selective Laser Melting Process. (2011). doi:10.26153/TSW/15290
68. Obeidi, M. A. *et al.* Laser beam powder bed fusion of nitinol shape memory alloy (SMA). *J. Mater. Res. Technol.* **14**, 2554–2570 (2021).
69. Lassègue, P. *et al.* Laser powder bed fusion (L-PBF) of Cu and CuCrZr parts: Influence of an absorptive physical vapor deposition (PVD) coating on the printing process. *Addit. Manuf.* **39**, 101888 (2021).
70. Yan, D. *et al.* Laser powder bed fusion and post processing of alloy 22. *Addit. Manuf.* **50**, 102490 (2022).
71. Baco-Carles, V. *et al.* Laser powder bed fusion applied to the manufacture of bulk or structured magnetic cores. *J. Mater. Res. Technol.* **18**, 599–610 (2022).
72. Qi, X., Takata, N., Suzuki, A., Kobashi, M. & Kato, M. Laser powder bed fusion of a near-eutectic Al–Fe binary alloy: Processing and microstructure. *Addit. Manuf.* **35**, 101308 (2020).
73. Stopyra, W., Gruber, K., Smolina, I., Kurzynowski, T. & Kuźnicka, B. Laser powder bed fusion of AA7075 alloy: Influence of process parameters on porosity and hot cracking. *Addit. Manuf.* **35**, 101270 (2020).
74. Zhang, Z. *et al.* Laser powder bed fusion of advanced submicrometer TiB2 reinforced high-performance Ni-based composite. *Mater. Sci. Eng. A* **817**, 141416 (2021).
75. Yang, T. *et al.* Laser powder bed fusion of AlSi10Mg: Influence of energy intensities on spatter and porosity evolution, microstructure and mechanical properties. *J. Alloys Compd.* **849**, 156300 (2020).
76. Han, Q. *et al.* Laser powder bed fusion of Hastelloy X: Effects of hot isostatic pressing and the hot cracking mechanism. *Mater. Sci. Eng. A* **732**, 228–239 (2018).
77. Lindström, V. *et al.* Laser Powder Bed Fusion of Metal Coated Copper Powders. *Materials (Basel).* **13**, 3493 (2020).
78. Yin, Y. *et al.* Laser powder bed fusion of Ni-based Hastelloy X superalloy: Microstructure, anisotropic mechanical properties and strengthening mechanisms. *Mater. Sci. Eng. A* **827**, 142076 (2021).
79. Criales, L. E. *et al.* Laser powder bed fusion of nickel alloy 625: Experimental investigations of effects of process parameters on melt pool size and shape with spatter analysis. *Int. J. Mach. Tools Manuf.* **121**, 22–36 (2017).
80. Li, J., Wu, Y., Zhou, B. & Wei, Z. Laser Powder Bed Fusion of Pure Tungsten: Effects of Process Parameters on Morphology, Densification, Microstructure. *Materials (Basel).* **14**, 165 (2020).

81. Paraschiv, A., Matache, G., Condruz, M. R., Frigioescu, T. F. & Pambaguian, L. Laser Powder Bed Fusion Process Parameters’ Optimization for Fabrication of Dense IN 625. *Materials (Basel).* **15**, 5777 (2022).
82. Miao, K. *et al.* Laser powder-bed-fusion of Si3N4 reinforced AlSi10Mg composites: Processing, mechanical properties and strengthening mechanisms. *Mater. Sci. Eng. A* **825**, 141874 (2021).
83. Chen, W. *et al.* Laser power modulated microstructure evolution, phase transformation and mechanical properties in NiTi fabricated by laser powder bed fusion. *J. Alloys Compd.* **861**, 157959 (2021).
84. Shuai, C. *et al.* Laser rapid solidification improves corrosion behavior of Mg-Zn-Zr alloy. *J. Alloys Compd.* **691**, 961–969 (2017).
85. Jadhav, S. D., Goossens, L. R., Kinds, Y., Hooreweder, B. Van & Vanmeensel, K. Laser-based powder bed fusion additive manufacturing of pure copper. *Addit. Manuf.* **42**, 101990 (2021).
86. Asherloo, M. *et al.* Laser-beam powder bed fusion of cost-effective non-spherical hydride-dehydride Ti-6Al-4V alloy. *Addit. Manuf.* **56**, 102875 (2022).
87. Nath, S. D., Kate, K., Irrinki, H. & Dilip, J. J. S. Laser-Powder Bed Fusion of 420 Stainless Steel for Mold and Surgical Tool Applications New Additive Manufacturing Paradigms for Low Cost Injection Molding Tools View project. (2017).
88. Cai, X., Malcolm, A. A., Wong, B. S. & Fan, Z. Measurement and characterization of porosity in aluminium selective laser melting parts using X-ray CT. *Virtual Phys. Prototyp.* **10**, 195–206 (2015).
89. Mehta, A. *et al.* Microstructural characteristics and mechanical properties of additively manufactured Cu–10Sn alloys by laser powder bed fusion. *Mater. Sci. Eng. A* **838**, 142775 (2022).
90. Nucci, S. Microstructural Development of Inconel 625 Nickel-Based Superalloy as Function of Laser Powder Bed Fusion Parameters. *Electronic Theses and Dissertations, 2020-* (2021).
91. Gangireddy, S., Gwalani, B., Liu, K., Faierson, E. J. & Mishra, R. S. Microstructure and mechanical behavior of an additive manufactured (AM) WE43-Mg alloy. *Addit. Manuf.* **26**, 53–64 (2019).
92. Zhou, L. *et al.* Microstructure and mechanical properties of Zr-modified aluminum alloy 5083 manufactured by laser powder bed fusion. *Addit. Manuf.* **28**, 485–496 (2019).
93. He, C. *et al.* Microstructure Evolution and Biodegradation Behavior of Laser Rapid Solidified Mg–Al–Zn Alloy. *Metals (Basel).* **7**, 105 (2017).
94. Kreitcberg, A., Brailovski, V. & Prokoshkin, S. New biocompatible near-beta Ti-Zr-Nb alloy processed by laser powder bed fusion: Process optimization. *J. Mater. Process. Technol.* **252**, 821–829 (2018).
95. Caiazzo, F., Alfieri, V. & Casalino, G. On the Relevance of Volumetric Energy Density in the Investigation of Inconel 718 Laser Powder Bed Fusion. *Materials (Basel).* **13**, 538 (2020).
96. Laohaprapanon, A. *et al.* Optimal Scanning Condition of Selective Laser Melting Processing with Stainless Steel 316L Powder. *Adv. Mater. Res.* **341**–**342**, 816–820 (2012).
97. Letenneur, M., Kreitcberg, A. & Brailovski, V. Optimization of Laser Powder Bed Fusion Processing Using a Combination of Melt Pool Modeling and Design of Experiment Approaches: Density Control. *J. Manuf. Mater. Process.* **3**, 21 (2019).
98. Andronov, V., Šimota, J., Beránek, L., Blažek, J. & Rušar, F. Optimization of Process Parameters for Additively Produced Tool Steel 1.2709 with a Layer Thickness of 100 μm. *Materials (Basel).* **14**, 2852 (2021).
99. Shi, C., Dietrich, S. & Schulze, V. Parameter optimization and mechanical properties of 42CrMo4 manufactured by laser powder bed fusion. *Int. J. Adv. Manuf. Technol.* **121**, 1899–1913 (2022).
100. Huynh, T. Parametric Investigation and Optimization for Inconel 718 Nickel-Based Superalloy in Laser Powder Bed Fusion. *Electronic Theses and Dissertations, 2020-* (2020).
101. Salarian, M., Asgari, H. & Vlasea, M. Pore space characteristics and corresponding effect on tensile properties of Inconel 625 fabricated via laser powder bed fusion. *Mater. Sci. Eng. A* **769**, 138525 (2020).

102. Vallejo, N. D. *et al.* Process Optimization and Microstructure Analysis to Understand Laser Powder Bed Fusion of 316L Stainless Steel. *Metals (Basel).* **11**, 832 (2021).
103. Yu, W. *et al.* Processing and characterization of crack-free 7075 aluminum alloys with elemental Zr modification by laser powder bed fusion. *Mater. Sci. Addit. Manuf.* **1**, 4 (2022).
104. Uddin, S. Z. *et al.* Processing and characterization of crack-free aluminum 6061 using high-temperature heating in laser powder bed fusion additive manufacturing. *Addit. Manuf.* **22**, 405–415 (2018).
105. Calignano, F., Manfredi, D., Marola, S., Lombardi, M. & Iuliano, L. Production of Dense Cu-10Sn Part by Laser Powder Bed Fusion with Low Surface Roughness and High Dimensional Accuracy. *Materials (Basel).* **15**, 3352 (2022).
106. Ozsoy, A., Yasa, E., Keles, M. & Tureyen, E. B. Pulsed-mode Selective Laser Melting of 17-4 PH stainless steel: Effect of laser parameters on density and mechanical properties. *J. Manuf. Process.* **68**, 910–922 (2021).
107. Aboulkhair, N. T., Everitt, N. M., Ashcroft, I. & Tuck, C. Reducing porosity in AlSi10Mg parts processed by selective laser melting. *Addit. Manuf.* **1**–**4**, 77–86 (2014).
108. Ghayoor, M. *et al.* Selective laser melting of 304L stainless steel: Role of volumetric energy density on the microstructure, texture and mechanical properties. *Addit. Manuf.* **32**, 101011 (2020).
109. Pawlak, A., Szymczyk, P. E., Kurzynowski, T. & Chlebus, E. Selective laser melting of magnesium AZ31B alloy powder. *Rapid Prototyp. J.* **26**, 249–258 (2020).
110. Sun, Z., Tan, X., Tor, S. B. & Yeong, W. Y. Selective laser melting of stainless steel 316L with low porosity and high build rates. *Mater. Des.* **104**, 197–204 (2016).
111. Zi, X. *et al.* Spheroidisation of tungsten powder by radio frequency plasma for selective laser melting. *Mater. Sci. Technol.* **34**, 735–742 (2017).
112. de Leon Nope, G. V. *et al.* Study of volumetric energy density limitations on the IN718 mesostructure and microstructure in laser powder bed fusion process. *J. Manuf. Process.* **64**, 1261–1272 (2021).
113. Neikter, M. *et al.* Tensile Properties of 21-6-9 Austenitic Stainless Steel Built Using Laser Powder-Bed Fusion. *Materials (Basel).* **14**, 4280 (2021).
114. Letenneur, M., Kreitcberg, A. & Brailovski, V. The Average Grain Size and Grain Aspect Ratio in Metal Laser Powder Bed Fusion: Modeling and Experiment. *J. Manuf. Mater. Process.* **4**, 25 (2020).
115. Alfaify, A. The Effect of Changing Particle Size Distribution and Layer Thickness on the Density of Parts Manufactured Using the Laser Powder Bed Fusion Process. (2019).
116. Georgilas, K., Khan, R. H. U. & Kartal, M. E. The influence of pulsed laser powder bed fusion process parameters on Inconel 718 material properties. *Mater. Sci. Eng. A* **769**, 138527 (2020).
117. Rebesan, P. *et al.* Tungsten Fabricated by Laser Powder Bed Fusion. *BHM Berg- und Hüttenmännische Monatshefte* **166**, 263–269 (2021).
118. Hyer, H. *et al.* Understanding the Laser Powder Bed Fusion of AlSi10Mg Alloy. *Metallogr. Microstruct. Anal.* **9**, 484–502 (2020).
