# Supplementary material for: Material-agnostic machine learning approach enables high relative density in powder bed fusion products
Source: Nat Commun. 2023 Oct 17;14:6557. doi: 10.1038/s41467-023-42319-x (PMC10582079; doi:10.1038/s41467-023-42319-x)
Supplement: Supplementary file 3 — Description of Additional Supplementary Files [file 41467_2023_42319_MOESM3_ESM.pdf]

Description of Additional Supplementary Files

File Name: Supplementary Data 1
Description: Prepared Dataset with 2167 process conditions and materials extracted from the literature. References can be found in the associated Supplementary Information.
